# Complete Genome Analysis of Highly Pathogenic Non-O1/O139 *Vibrio cholerae* Isolated From *Macrobrachium rosenbergii* Reveals Pathogenicity and Antibiotic Resistance-Related Genes

**DOI:** 10.3389/fvets.2022.882885

**Published:** 2022-05-18

**Authors:** Yifan Zhou, Shuwen Gu, Jie Li, Peng Ji, Yingjie Zhang, Congcong Wu, Qun Jiang, Xiaojian Gao, Xiaojun Zhang

**Affiliations:** College of Animal Science and Technology, Yangzhou University, Yangzhou, China

**Keywords:** non-O1/O139 *Vibrio cholerae*, genomic characterization, virulence-related genes, antibiotic resistance-related genes, secretion systems

## Abstract

Non-O1/O139 *Vibrio cholerae* is a highly virulent pathogen that causes mass mortalities of various aquatic animals. In the present study, we sequenced the whole genome of non-O1/O139 *V*. *cholerae* GXFL1-4, isolated from *Macrobrachium rosenbergii*, to reveal the pathogenicity and antibiotic resistance. The result showed its genome contained two circular chromosomes and one plasmid with a total size of 4,282,243 bp, which harbored 3,869 coding genes. Among them, 3,047, 2,659, and 3,661 genes were annotated in the Clusters of Orthologous Genes (COG), Gene Ontology (GO), and Kyoto Encyclopedia of Genes and Genomes (KEGG), respectively. In addition, 372 potential virulence genes were predicted based on the Virulence Factor Database (VFDB) database, such as type II, III, IV, and VI secretion systems related genes, flagella genes, and pilus formation or motility-related genes. Blast results in the Comprehensive Antibiotic Resistance Database (CARD) database showed that the strain contained 148 antibiotic resistance-related genes belonging to 27 categories, such as efflux pump complex antibiotic resistance genes and antibiotic resistance gene cluster genes. The Pathogen-Host Interaction (PHI) database annotated 320 genes related to pathogen-host interaction, such as T3SS, virulence regulatory factors, transcriptional regulators, and two-component response regulator related genes. The whole-genome analysis suggested that the pathogenic non-O1/O139 *V. cholerae* strain GXFL1-4 might have a complex molecular mechanism of pathogenicity and antibiotic resistance. This study provides a wealth of information about non-O1/O139 *V. cholerae* genes related to its pathogenicity and drug resistance and will facilitate the understanding of its pathogenesis as well as the development of prevention and treatment strategies for the pathogen.

## Introduction

*Vibrio cholerae* is a Gram-negative bacterium with more than 200 serogroups, which do not possess the cholera toxin, and the toxin-coregulated pilus virulence factors are commonly designated as non-O1/O139 *V. cholerae*. Non-O1/O139 *V. cholerae* is an important pathogen of zoonosis that is ubiquitously distributed in aquatic environments (1). With the intensification of aquaculture, non-O1/O139 *V. cholerae* has become one of the most serious problems to endanger sustainable aquaculture development (2). The non-O1/O139 *V. cholerae* caused high mortality and great economic loss in a variety of aquatic animals, such as *Penaeus vannamei, Macrobrachium nipponense, Cyprinus carpio*, and *Carassius auratus* (3–5). For the great losses caused by non-O1/O139 *V. cholerae* to the aquaculture industry, our previous studies revealed that non-O1/O139 *V. cholerae* GXFL1-4 is a highly virulent strain isolated from *M. rosenbergii* that causes mass mortalities in *M. rosenbergii* (6). Despite these prior studies, the pathogenic mechanism of non-O1/O139 *V. cholerae* during infection in aquatic animals is poorly understood.

Recent vigoros complete genome analysis has been performed to unravel putative strain-specific virulence determinants. Complete genome analysis of pathogenic bacteria provides a powerful tool for better understanding the mechanisms of bacterial pathogenicity and their drug-resistant properties, which provide the new genetic and molecular approaches for controlling the disease (7). Whole-genome sequencing has been increasingly employed to analyze the relationship among the aquatic animals, the environment, and the virulence of strains (8, 9). At present, there are some complete genome sequences of *V. cholerae* from humans and environments in the Genbank genome database. However, the genetic features and evolutionary strategies of non-O1/O139 *V. cholerae* from aquatic animals remain largely unknown.

In this study, the complete genome of highly pathogenic non-O1/O139 *V. cholerae* strain GXFL1-4, isolated from the diseased *M. rosenbergii*, was sequenced, and the genes related to pathogenicity, drug resistance, and host–pathogens interaction were identified. This research will provide valuable insights into the prevention and treatment of diseases caused by non-O1/O139 *V. cholerae*.

## Materials and Methods

### Bacteria, Library Construction, and Sequencing

A non-O1/O139 *V. cholerae* GXFL1-4 was isolated from diseased *M. rosenbergii* at a commercial aquaculture farm in Gaoyou County, Jiangsu Province, China. Challenges showed that bath immersion of strain GXFL1-4 caused red body syndrome and mass mortalities of *M. rosenbergii* (6). The strain was inoculated into 100 ml of LB broth medium and incubated at 28°C with shaking at 200 rpm for 18 h. The culture was then centrifuged at 10,000 rpm for 2 min to pellet the cells. The collected cells were then washed and re-suspended in sterile 1 × phosphate-buffered saline (PBS). From this, 1 ml aliquots were collected in microcentrifuge tubes and used for further studies.

Lim et al. used the sodium dodecyl sulfate (SDS) method to extract genomic DNA (10). The harvested DNA was detected by the agarose gel electrophoresis and quantified by the Qubit® 2.0 Fluorometer (Thermo Scientific). A total amount of 1 μg DNA per sample was used as input material for the DNA sample preparations. Sequencing libraries were generated using the NEBNext® Ultra™ DNA Library Prep Kit for Illumina (NEB, USA) following the manufacturer's recommendations and index codes were added to attribute sequences to each sample. The whole genome of GXFL1-4 was sequenced using Illumina NovaSeq PE150 at the Beijing Novogene Bioinformatics Technology Co., Ltd.

### Genome Assembly

Clean data are assembled with Short Oligonucleotide Alignment Program SOAP denovo software (11), SPAdes software (12), and Assembly By Short Sequences (Abyss) software (13). The assembly results of the three software were integrated with CISA software and the assembly result with the least scaffolds was selected. The GapCloser software was used to fill the gap in the preliminary assembly results. The same lane pollution by filtering the reads with low sequencing depth was removed to obtain the whole genome.

### Genome Component Prediction

For bacteria, we used the GeneMarkS (Version 4.17) program to retrieve the related coding gene (14). The interspersed repetitive sequences were predicted using the RepeatMasker (Version open-4.0.5) (15). Transfer RNA (tRNA) genes were predicted by the tRNAscan-SE (Version 1.3.1) (16, 17). Ribosome RNA (rRNA) genes were analyzed by the rRNAmmer (Version 1.2). Small nuclear RNAs (snRNA) were predicted by BLAST against the Rfam database (Version 1.1rc4) (18, 19). The IslandPath-DIOMB program was used to predict the Genomics Islan (Version 0.2ds) (20).

### Gene Function

We used different databases to predict gene functions, which were Gene Ontology (GO) (http://www.geneontology.org) (21), Kyoto Encyclopedia of Genes and Genomes (KEGG) (http://www.genome.jp/kegg) (22), and Clusters of Orthologous Groups (COG) (http://www.ncbi.nlm.nih.gov/COG/) (23), respectively. The prediction of Type I–VII proteins secreted by the pathogenic bacteria was based on the Effective T3 software (Version 1.0.1). For the pathogenicity and antibiotic resistance of pathogenic bacteria, we used the Pathogen Host Interactions (PHI) (http://www.phi-base.org/index.jsp) (24), Virulence Factors of Pathogenic Bacteria (VFDB) (http://www.mgc.ac.cn/VFs) (25), and Comprehensive Antibiotic Research Database (CARD) (http://arpcard.mcmaster.ca) (26) to perform the above analyses. A whole-genome BLAST search (E-value less than 1e-5, minimal alignment length percentage larger than 40%) was conducted against the above databases.

### Antibiotic-Resistant Test

The sensitivity of strain GXFL1-4 to 36 antibiotics was evaluated by disc diffusion method (27) on Muller-Hinton agar using commercial antibiotic discs. All the tests were performed in triplicate. The antibiotic sensitivity of the strains as resistant, intermediate, or sensitive was measured by the inhibition zone diameter according to standards suggested by Hangzhou Tianhe Microorganism Reagent Co.

## Results

### Genome Structure and General Features

**Figure 1:**
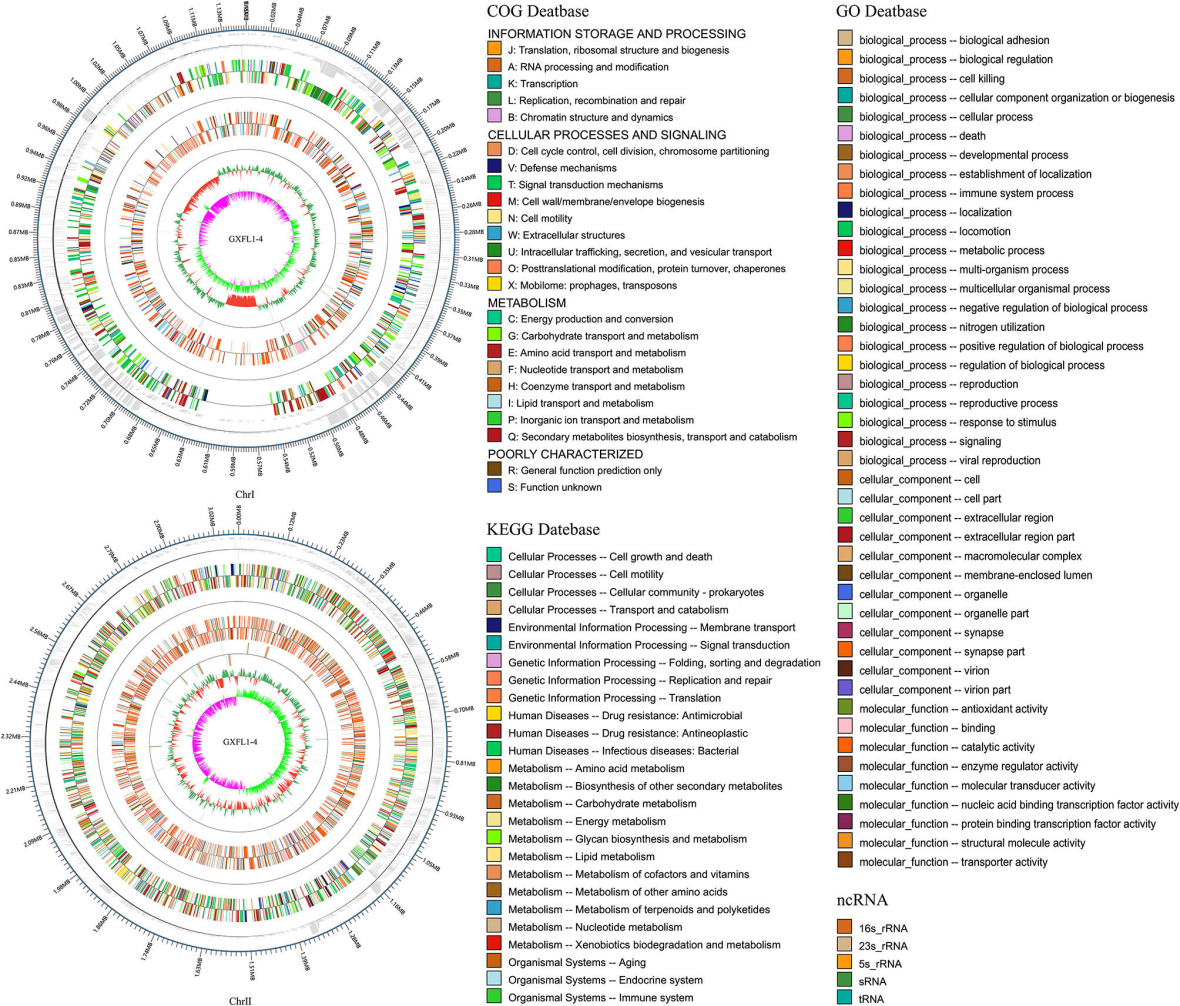
Circular genome map of non-O1/O139 *Vibrio cholerae* GXFL1-4.

The genome of non-O1/O139 *V. cholerae* strain GXFL1-4 was sequenced using Nanopore, and the genome characteristics are summarized in Table 1. After filtering, all reads were assembled into two circular chromosomes and one circular plasmid (Figure 1). Chromosome I and II are 1,154,802 and 3,079,090 bp, with a G + C content of 45.99 and 47.97%, respectively (NCBI accession number CP090386 and CP090387). The plasmid is 48,351 bp, with a G+C content of 41.3% (NCBI accession number CP090388). Table 1 and Figure 1 show the annotation information of general features.

**Table 1:** Genomic information for non-O1/O139 *Vibrio cholerae* strain GXFL1-4.

	**Chromosome I**	**Chromosome II**	**Plasmid**
Size (bp)	1,154,802	3,079,090	48,351
C + G content (%)	45.99	47.97	41.3
CDS	1,080	2,722	67
Pseudogenes or gene fragments	278	387	37
Genes assigned to COGs	758	2,282	7
Average CDS length	864.19	947.37	658.5
rRNA genes	0	31	0
tRNA genes	4	99	0

### COG Analysis

**Figure 2:**
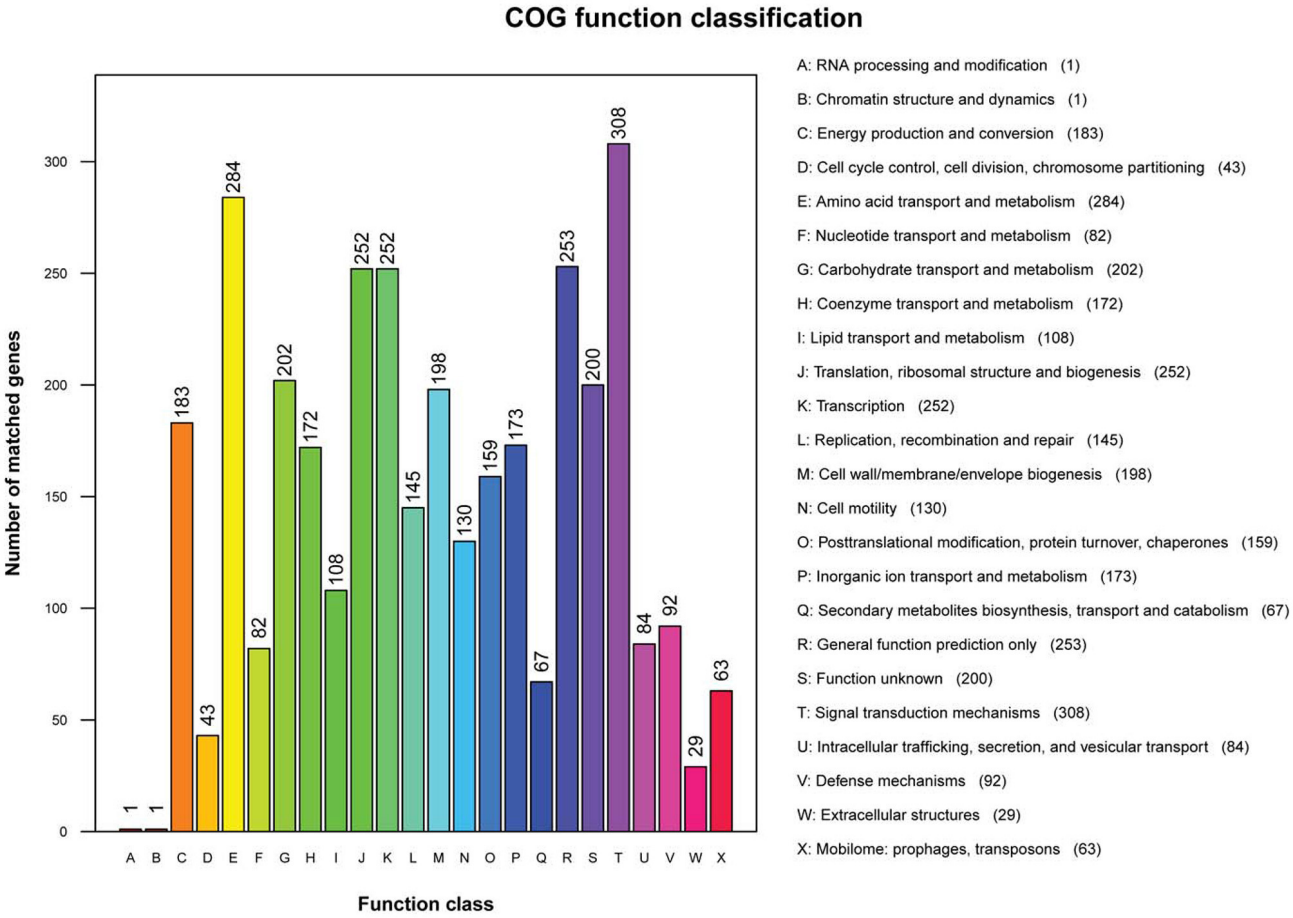
The Clusters of Orthologous Genes (COG) functional annotation in the whole genome of non-O1/O139 *V. cholerae* strain GXFL1-4.

Clusters of Orthologous Groups annotation results showed that 3,047 genes were annotated into 24 classes of genes, accounting for 78.75% of total genes in non-O1/O139 *V. cholerae* strain GXFL1-4. The number of each class of gene was as follows: RNA processing and modification gene (1), chromatin structure and dynamics gene (1), energy production and conversion genes (183), cell cycle control, cell division, and chromosome partitioning genes (43), amino acid transport and metabolism genes (284), nucleotide transport and metabolism genes (82), carbohydrate transport and metabolism genes (202), coenzyme transport and metabolism genes (172), lipid transport and metabolism genes (108), translation, ribosomal structure, and biogenesis genes (252), transcription genes (252), replication, recombination, and repair genes (145), cell wall/membrane/envelope biogenesis genes (196), cell motility genes (130), posttranslational modification, protein turnover, chaperones genes (159), inorganic ion transport and metabolism genes (173), secondary metabolites biosynthesis, transport, and catabolism genes (67), general function prediction only genes (253), function unknown genes (200), signal transduction mechanisms genes (308), intracellular trafficking, secretion, and vesicular transport genes (84), defense mechanisms genes (92), extracellular structures genes (28), and mobilome: prophages and transposons genes (63) (Figure 2). Supplementary Table 1 lists all COG functional annotation information.

### GO Analysis

**Figure 3:**
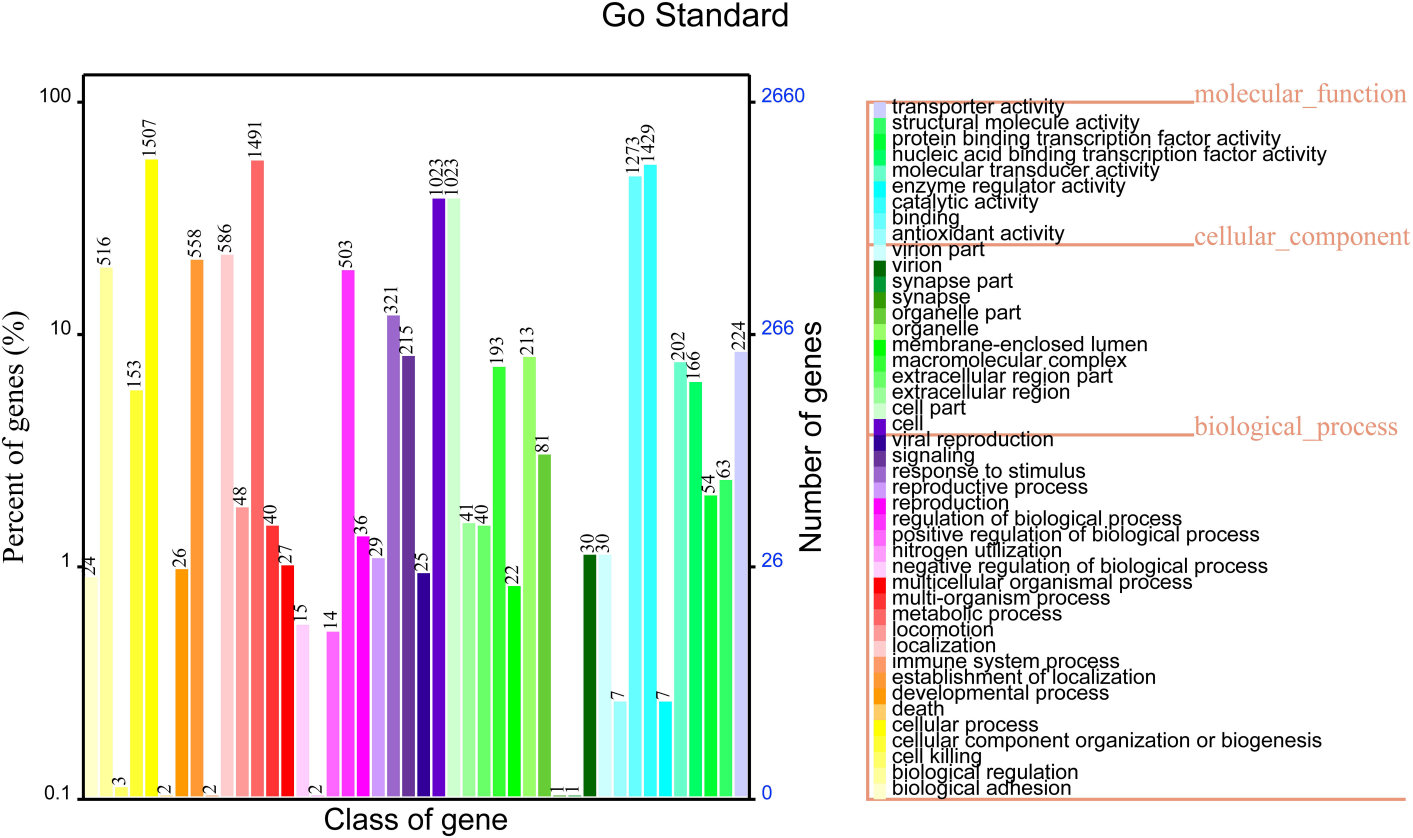
Gene Oncology (GO) functional annotation in the whole genome of non-O1/O139 *V. cholerae* strain GXFL1-4.

The functional annotation results in the GO database showed that 2,659 genes were annotated into three classes of genes, which accounted for 68.72% of total genes of non-O1/O139 *V. cholerae* strain GXFL1-4. Among them, 1,041 genes were related to the cellular component, 2,142 genes were related to the molecular function, and 2069 genes were related to the biological process. The highest numbers of genes in the classification of the biological process were biological adhesion process (1,507) and biological regulation (1,491). The highest numbers of genes in the classification of cellular components were cell (1,023), cell part(1,023), extracellular region (213), and extracellular region part (193). The highest numbers of genes in the classification of molecular function were antioxidant activity (1,429) and binding (1,273). More information is shown in Figure 3. Supplementary Table 2 lists all GO functional annotation information.

### KEGG Analysis

**Figure 4:**
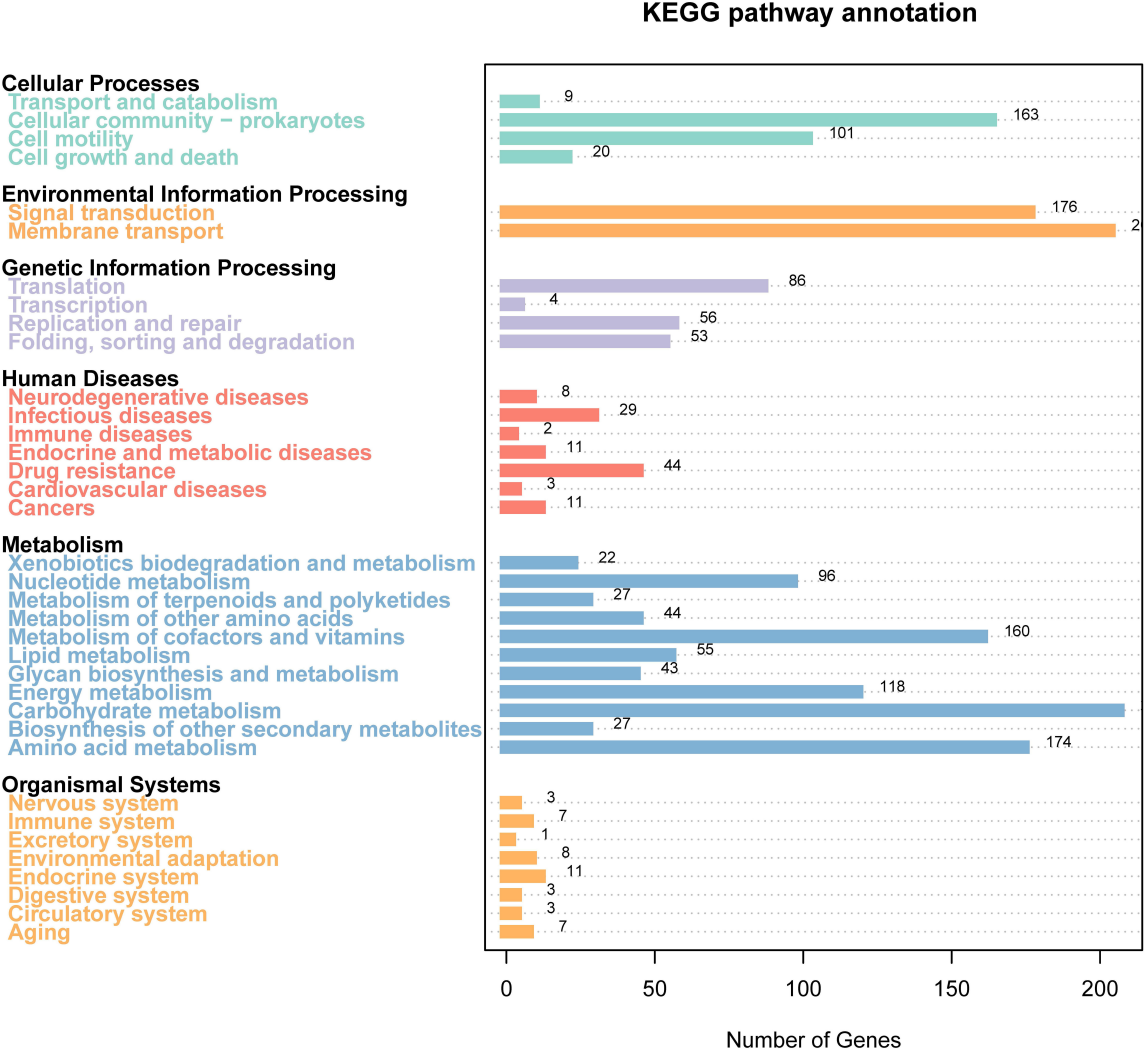
Gene distribution based on the Kyoto Encyclopedia of Genes and Genomes (KEGG) classification of non-O1/O139 *V. cholerae* GXFL1-4.

The results of the KEGG pathway analysis showed that 3,661 genes were annotated into 189 known metabolic pathways. Among them, the largest number of genes was ABC transporters (103), followed by the two-component system (101), biofilm formation (83), ribosome (54), and quorum sensing (48). Cluster analysis showed that 196 metabolic pathways were categorized into six classifications of cellular processes, metabolism, human diseases, genetic information processing, organismal systems, and environmental information processing, and the numbers of genes in these six classifications were 215, 681, 78, 184, 37, and 275, respectively (Figure 4). The 215 genes in the classification of cellular processes could be divided into four categories, and most of them were clustered into the cellular community—prokaryotes (173) and cellular community-prokaryotes (52). The 681 genes in the classification of metabolism were divided into 11 categories. The categories included carbohydrate metabolism (287), amino acid metabolism (205), metabolism of cofactors and vitamins (159), and energy metabolism (127). The 78 genes in the classification of human diseases were clustered into 7 categories, and the categories of the largest gene number were antibiotic resistance: drug resistance (44), infectious diseases (22), and neurodegenerative diseases (14). The 184 genes in the classification of genetic information processing were clustered into three categories. The categories of the largest gene numbers were transcription (85), replication and repair (81), and folding, sorting, and degradation (49). The 37 genes in the classification of organismal systems were divided into eight categories. Among them, the top three categories with the largest gene numbers were the endocrine system (11), aging (7), immune system (6), and nervous system (5). The environmental information was divided into two categories, such as membrane transport (162) and signal transduction (115). Supplementary Table 3 shows all KEGG pathway annotation information.

### Prediction of Virulence Genes of the Strains GXFL1-4

A total of 372 potential virulence genes were predicted in the whole genome of the strain GXFL1-4. Moreover, 174 genes had annotation information in VFDB database that included 143 offensive virulence genes, 22 defensive virulence genes, 6 virulence-associated regulation genes, and 3 non-specific virulence genes (Table 2). Supplementary Table 4 shows detailed VFDB annotation information.

**Table 2:** The annotation of virulence factors of the strain GXFL1-4 in VFDB databases.

**Virulence primary categories**	**Virulence Secondary categories**	**Gene numbers**
Offensive virulence factors	Adherence	42
Offensive virulence factors	Invasion	69
Offensive virulence factors	Secretion system	22
Offensive virulence factors	Toxin	10
Defensive virulence factors	Antiphagocytosis	13
Defensive virulence factors	Serum resistance	1
Defensive virulence factors	Stress protein	7
Non-specific virulence factor	Iron uptake system	3
Regulation of virulence-associated genes	Regulation	6

Among the offensive virulence genes, there were 42 genes related to adherence, including pilus formation or motility genes, such as *mshB/C/D, mshE/F/G*, membrane protein *ompU*, and multivalent adhesion molecule *MAM7*. There were 69 genes related to invasion, including flagella formation or motility exercise-related genes, such as *flaA, fleQ, flgB, flaG, flhA, fliG, fliM, motA*, transcriptional regulator *fleQ, flrA*, chemotaxis protein, *cheA*, and *cheR*.

### Analysis of Antibiotic Resistance

A total of 36 antibiotics belonging to 12 categories were selected to test the antimicrobial phenotype of strain GXFL1-4 through the Kirby-Bauer disk diffusion method (Table 3). It was sensitive to cefoxitin, streptomycin, tobramycin, erythromycin, spectinomycin, chloramphenicol, amikacin, tetracycline, polymyxin B, and minocycline.

**Table 3:** Antibiotic resistance of non-O1/O139 *V. cholera* GXFL1-4.

**Antibiotics**	**Disccontent (μg)**	**Inhibition zone diameter (mm)**
Ampicillin	10	0 (R)
Aztreonam	30	9 ± 0.12 (R)
Cefazolin	30	0 (R)
Cephalothin	30	0 (R)
Cefuroxime	30	0 (R)
Cefoperazone	75	9 ± 0.14 (R)
Cefotaxime	30	0 (R)
Ceftriaxone	30	0 (R)
Cefepime	30	0 (R)
Ceftazidime	30	17 ± 0.15 (I)
Cefoxitin	30	19 ± 0.12 (S)
Fleroxacin	10	14 ± 0.14 (I)
Streptomycin	10	15 ± 0.10 (S)
Tobramycin	10	17 ± 0.11 (S)
Kanamycin	30	14 ± 0.08 (I)
Midecamycin	30	15 ± 0.09 (S)
Piperacillin	100	0 (R)
Gentamicin	10	18 ± 0.10 (S)
Penicillin	10	0 (R)
Erythromycin	15	22 ± 0.09 (S)
Spectinomyci	100	22 ± 0.13 (S)
Clarithromycin	15	17 ± 0.10 (I)
Clindamycim	2	0 (R)
Chloramphenicol	30	22 ± 0.11 (S)
Amikacin	30	17 ± 0.10 (S)
Tetracycline	30	21 ± 0.09 (S)
Minocycline	30	23 ± 0.08 (S)
Nofloxacin	10	13 ± 0.17 (I)
Ciprofloxacin	2	16 ± 0.15 (I)
Levofloxacin	5	13 ± 0.13 (R)
Polymyxin B	300	13 ± 0.18 (S)
Paediatric Compound Sulfamethoxazole Tablets	23.75	0 (R)
Nitrofurantoin	300	0 (R)
Florfenicol	5	21 ± 0.11 (S)
Enrofloxacin	5	13 ± 0.19 (I)
Oxacillin	1	0 (R)

“*S”, sensitive; “I”, intermediate; and “R”, resistance*.

The results of the CARD database showed that 148 antibiotic resistance genes belonging to 27 categories were found in the whole genome of the strain GXFL1-4 (Table 4). In terms of antibiotic resistance phenotype and genotype correlation, the strain GXFL1-4 contained the penam-resistant genes (*evgS, adeL, CRP, NmcR, mecC*, and *smeR*), cephalosporin-resistant genes (*NmcR, CTX-M-99*, and *smeR*), monobactam-resistant genes (*OpmB*), quinolone-resistant genes (*evgS, adeL, patA, arlR, mfd, MexF*/*V*/*I, CRP, cdeA, QnrVC5, emrB*, and *hmrM*), sulfonamide-resistant genes (*sul2/3*), and lincosamide-resistant genes [*msrB, RlmA*(II), and *clbB*]. The phenotype was consistent with the genotype. In addition, multidrug efflux pump systems and two-component regulatory systems were also found in the strain. Supplementary Table 5 shows antibiotic resistance genes additional information.

**Table 4:** The antibiotic resistance genes of the strain GXFL1-4 annotated in CARD.

**Categories**	**Gene numbers**	**Gene name**
pleuromutilin antibiotic	8	*TaeA, msrB, clbB*
carbapenem	11	*NmcR*
sulfonamide antibiotic	2	*sul2, sul3*
aminoglycoside antibiotic	9	*cpxA, baeR, acrD, APH(3”)-Ib, APH(6)-Id, kdpE, smeR*
macrolide antibiotic	41	*macB, evgS, adeL, msrB, MexV, CRP, RlmA(II), MexJ, OpmB, MexK*
glycopeptide antibiotic	10	*vanRI, vanTrL, vanTG, vanRB, vanRG, vanHB, vanE, vanHD, vanHO*
tetracycline antibiotic	38	*evgS, adeL, msrB, tetT, tet(35), tcr3, MexV, MexI, tet34, tetB(60), emrK, otr(A), MexJ, OpmB, MexK*
monobactam	1	*OpmB*
diaminopyrimidine antibiotic	3	*dfrA3, dfrA17, MexF*
streptogramin antibiotic	4	*msrB, clbB*
glycylcycline	1	*TriC*
aminocoumarin antibiotic	6	*cpxA, baeR, OpmB*
cephamycin	2	*NmcR, smeR*
penem	3	*NmcR*
phenicol antibiotic	9	*msrB, catB9, MexV, clbB, MexF*
rifamycin antibiotic	2	*rphB, iri*,
triclosan	3	*TriC, MexJ, MexK*
acridine dye	11	*arlR, leuO, MexV, MexI, cdeA, hmrM*
peptide antibiotic	15	*PmrE, vanRI, vanTrL, rosB, vanTG, bacA, vanRB, vanRG, arnA, vanHB, basS, MCR-3, vanE, vanHD, vanHO*
lincosamide antibiotic	5	*msrB, clbB, RlmA(II)*
fluoroquinolone antibiotic	38	*evgS, adeL, patA, arlR, mfd, MexV, MexI, CRP, cdeA, QnrVC5, emrB, hmrM, MexF*
nitroimidazole antibiotic	1	*msbA*
penam	21	*evgS, adeL, CRP, NmcR, mecC, smeR*
cephalosporin	3	*NmcR, CTX-M-99, smeR*
bicyclomycin	3	*bcr-1*
nucleoside antibiotic	2	*leuO*
oxazolidinone antibiotic	4	*msrB, clbB*

### Genomic Islands Analysis

**Figure 5:**
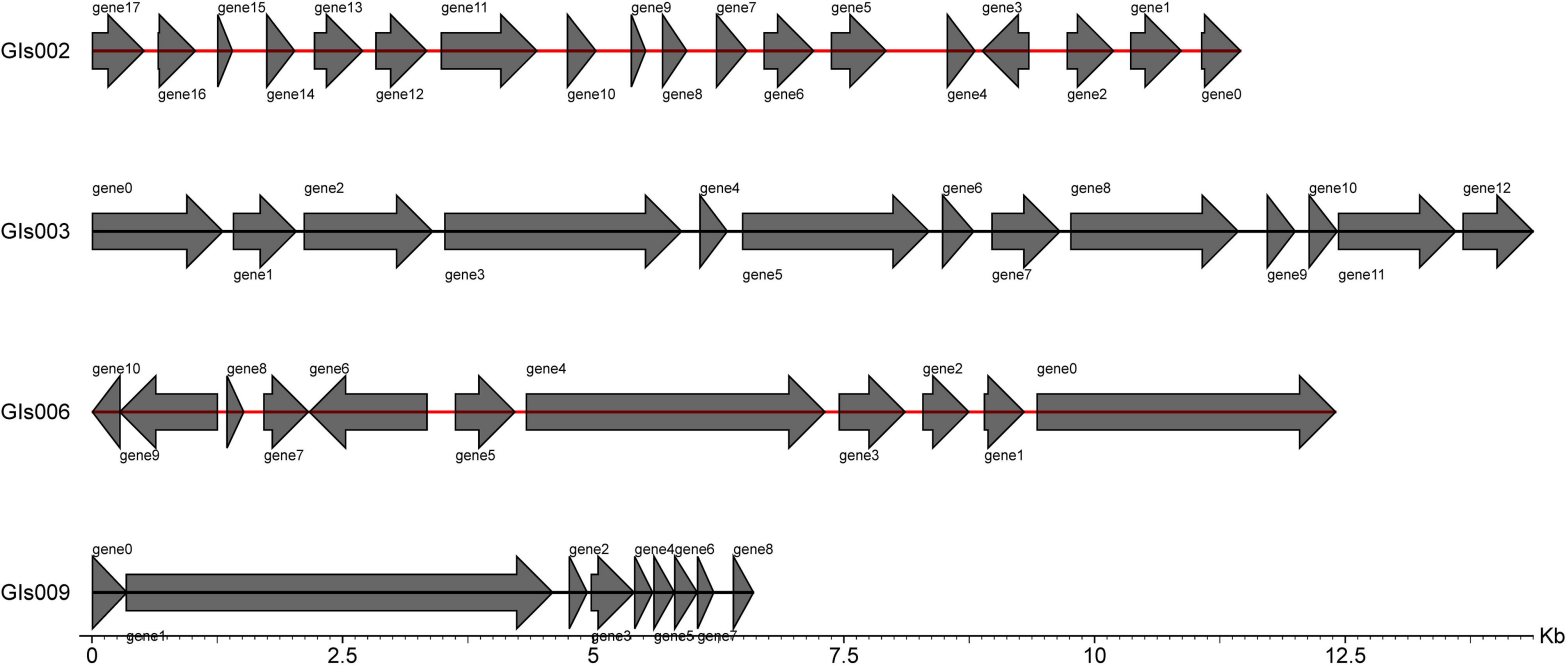
Statistical map of gene distribution in genomic islands.

Through the IslandPath-DIOMB online system, 9 genomic islands were predicted to be contained in the whole genome of the strain GXFL1-4 (Figure 5). The longest genomic island was 81,279 bp, and the shortest one was 6,594 bp. Supplementary Table 6 illustrates their detailed information.

### Predictive Analysis of Pathogen-Host Interaction Between the Strain GXFL1-4 and Host

**Figure 6:**
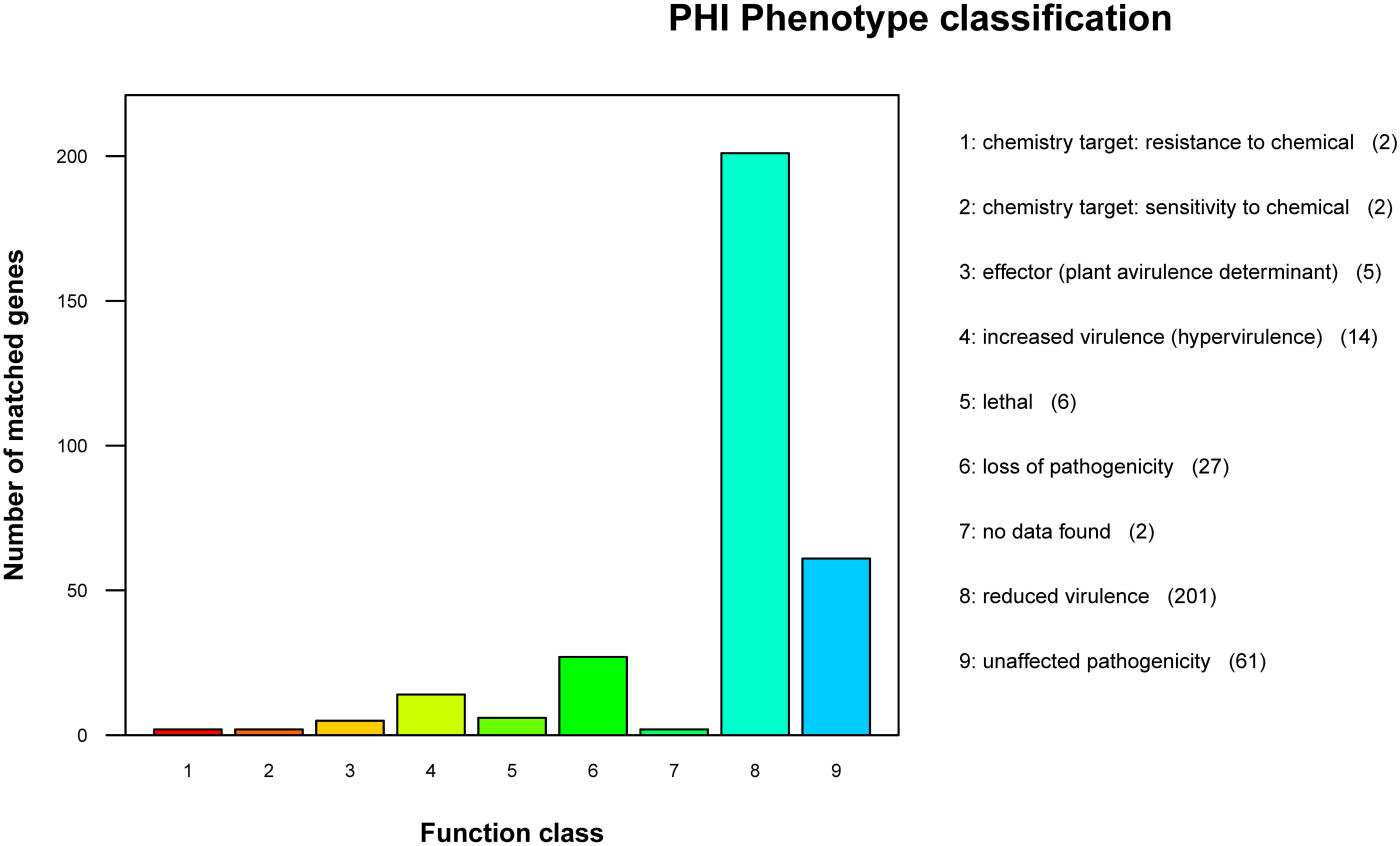
Pathogen-Host Interaction (PHI) phenotype mutation type distribution map.

According to the annotation results of the PHI database (Figure 6), the strain GXFL1-4 contained 320 genes related to PHI. Among them, there were 201 genes related to reduced virulence, 27 genes related to loss of pathogenicity, 14 genes related to hypervirulence, 6 genes related to lethal factors, 5 genes related to effectors, 2 genes related to chemical resistance, 2 genes related to chemical sensitivity, and 61 genes related to unaffected pathogenicity. The key genes associated with high pathogenicity were hypervirulence and lethal genes. Among them, such as T3SS function genes: *Ndk, GroEL, Rv2220*, and *fabG1*; virulence regulatory factors: *raxQ, raxP*, and *kdpE*; transcriptional regulators, *aphB* and *ohrR*; and two-component response regulator, *LuxO, arcA*, and *CpxR*.

### Secretory System Analysis

In this study, type II, type III, type IV, and type VI secretory systems were represented by genes in the GXFL1-4 genome. Moreover, 13 genes were assigned to types II, 155 genes were assigned to type III, two genes were assigned to type VI, and one gene was assigned to type VI. The genes assigned to the type II secretory systems were all present on chromosome II. The genes assigned to the type IV secretory systems were present on plasmid I. The genes assigned to the type VI secretory systems were present on chromosome I. In non-O1/O139 *V. cholerae* strain GXFL1-4, type III secretory system related genes were encoded on both chromosomes I and II, as well as on the plasmid.

## Discussions

In this study, we found that the whole genome of pathogenic non-O1/O139 *V. cholerae* strain GXFL1-4 was 4,282,243 bp in length, including two circular chromosomes and one plasmid, which was consistent with the genomes of *V. cholera* (29). In the gene annotation analysis, a large number of genes were annotated in the KEGG pathways, which are related to human diseases. Previous studies also showed that non-O1/O139 *V. cholerae* can cause liver disease, lung disease, gastroenteritis, and so on (28, 30, 31), which demonstrates the potential threat of non-O1/O139 *V. cholerae* strain GXFL1-4 to humans.

Extracellular products play an important role in the pathogenesis of various aquatic pathogens. In this study, the prediction of virulence-related genes showed that non-O1/O139 *V. cholerae* GXFL1-4 contains caseinase-related genes (*Vsp*), lipase-related genes (*YplA*), lecithinase–related genes (*VvPlpA*), and hemolysin-related genes (*HlyA*) in the whole genome. Previous studies have confirmed that the above extracellular products can cause harm to aquatic animals (6, 32). In addition, a number of genes coding motility and synergistic factors have been annotated, such as the flagella-related genes (*flaA, fliD, flgB*), chemotaxis factors (*cheA, cheB, cheY*), pili-related genes (*pilE, fimT, mshB*), and outer membrane proteins-related genes (*OmpU*). These genes were responsible for colonizing the host through movement and adhesion, finally causing the host disease (33, 34). These results indicated that non-O1/O139 *V. cholerae* carries many virulence genes, which might be important for pathogenicity.

It is generally believed that proteins and toxins of pathogenic bacteria are related to secretory systems. In this study, the type II, type III, type IV, and type VI secretory systems-related genes were predicted in the non-O1/O139 *V. cholerae* GXFL1-4 genome. In the genome data, the type II pathway components are encoded by 12 extracellular protein secretion (eps) genes and one mannose-sensitive hemagglutinin (MSHA) pili gene. These genes support proteins and toxins to cross the outer membrane, its process including translocation across the cytoplasmic membrane and the outer membrane (35). Often, the type II secretory system works along with other secretion systems to achieve full virulence. The type III secretory system promotes the transfer of bacterial effector proteins to the cytoplasm or the plasma membrane of target eukaryotic cells. In type III secretory system, a large number of flagellin genes, flagellar-related genes, and chemotaxis protein-related genes are annotated, which promote bacterial invasion and colonization, subvert host cells (36). The type IV secretory system is the most ubiquitous secretion system in nature, which annotated gene *TraK* can mediate the translocation of DNA or effector molecules into bacterial and eukaryotic target cells (37). The type VI secretory system is a cell envelope spanning machine in which the related gene *VgrG* translocates toxic effector proteins into eukaryotic and prokaryotic cells and has a pivotal role in pathogenesis and bacterial competition (38).

Multiple antibiotic resistance genes were identified in our predictive analysis of antibiotic resistance genes. Among them, activation of the *CpxA* system stimulates *mar* transcription, indicating that it can enhance the multidrug resistance cascade (39). Functional studies have shown that *MsbA* also is a polyspecific transporter capable of recognizing and transporting a wide spectrum of drug molecules (40). Moreover, multidrug efflux pumps are thought to be involved in mediating multidrug resistance in pathogenic bacteria. *OpmB* gene products showed functional cooperation with a membrane fusion protein of the *MexVW*-*OprM* multidrug efflux complex, *MexV*, a channel that is formed through both the inner and outer membrane, which can mediate the removal of xenobiotics, such as antibiotics, responding for resistance to antimicrobial agents (41). These results showed that the resistance mechanism of the strain GXFL1-4 might be complex.

## Data Availability Statement

The datasets presented in this study can be found in online repositories. The names of the repository/repositories and accession number(s) can be found in the article/Supplementary Material.

## Author Contributions

YZho, SG, JL, PJ, and YZha contributed to conception and design of the study. YZho organized the database. CW performed the statistical analysis. QJ wrote the first draft of the manuscript. XG and XZ wrote sections of the manuscript. All authors contributed to manuscript revision, read, and approved the submitted version.

## Funding

This work was supported by the National Natural Science Fund (31972830), the earmarked fund for Jiangsu Agricultural Industry Technology System (JATS [2021] 501, JATS [2021] 505), the National Key Research and Development Project (2019YFD0900305), and Revitalizing of Seed Industry—the Open Competition Mechanism to Select the Best Candidates Projects (JBGS [2021] 120). This work was generously supported by a studentship from the Yangzhou University.

## Conflict of Interest

The authors declare that the research was conducted in the absence of any commercial or financial relationships that could be construed as a potential conflict of interest.

## Publisher's Note

All claims expressed in this article are solely those of the authors and do not necessarily represent those of their affiliated organizations, or those of the publisher, the editors and the reviewers. Any product that may be evaluated in this article, or claim that may be made by its manufacturer, is not guaranteed or endorsed by the publisher.
